# Identification and Validation of Prognostic Genes Related to Histone Lactylation Modification in Glioblastoma: An Integrated Analysis of Transcriptome and Single-cell RNA Sequencing

**DOI:** 10.7150/jca.110646

**Published:** 2025-03-21

**Authors:** Wenfeng He, Ruihong Chen, Guangliang Chen, Lihan Zhang, Yuhang Qian, Jie Zhou, Jianhua Peng, Vincent Kam Wai Wong, Yong Jiang

**Affiliations:** 1 Dr. Neher's Biophysics Laboratory for Innovative Drug Discovery & State Key Laboratory of Quality Research in Chinese Medicine & Faculty of Chinese Medicine, Macau University of Science and Technology, Macau, China.; 2 Faculty of Chinese Medicine, Macau University of Science and Technology, Macau, China.; 3 Department of Oncology, The Affiliated Hospital of Southwest Medical University, Luzhou, China.; 4 Department of Neurosurgery, The Affiliated Hospital of Southwest Medical University, Luzhou, China.; 5 Guangdong Provincial Key Laboratory of Medical Molecular Diagnostics, The First Dongguan Affiliated Hospital, Guangdong Medical University, Dongguan, China.; 6 Songshan Lake Innovation Center of Medicine & Engineering, Guangdong Medical University, Dongguan, China.; 7 Sichuan Clinical Research Center for Neurosurgery, The Affiliated Hospital of Southwest Medical University, Luzhou, China.; 8 Institute of Epigenetics and Brain Science, Southwest Medical University, Luzhou, China.; 9 Academician (Expert) Workstation of Sichuan Province, The Affiliated Hospital of Southwest Medical University, Luzhou, China.; 10 Laboratory of Neurological Diseases and Brain Function, The Affiliated Hospital of Southwest Medical University, Luzhou, China.

**Keywords:** Glioblastoma, Histone lactylation modification, Prognostic genes, Single-cell RNA sequencing

## Abstract

**Background:** The impact of histone lactylation modification (HLM) on glioblastoma (GBM) progression is not well understood. This study aimed to identify HLM-associated prognostic genes in GBM and explore their mechanisms of action.

**Methods:** The presence and role of lactylation in glioma clinical tissue samples and its correlation with GBM progression were analysed through immunohistochemical staining and Western blotting. Sequencing data for GBM were obtained from publicly available databases. An initial correlation analysis was performed between global HLM levels and GBM. Differentially expressed HLM-related genes (HLMRGs) in GBM were identified by intersecting differentially expressed genes (DEGs) from the TCGA-GBM dataset, key module genes derived from weighted gene coexpression network analysis (WGCNA), and previously reported HLMRGs. Prognostic genes were subsequently identified through univariate Cox regression and least absolute shrinkage and selection operator (LASSO) regression analyses, which formed the basis for constructing a risk prediction model. Associations between HLMRGs and GBM were further evaluated via single-cell RNA sequencing (scRNA-seq) datasets. Complementary analyses, including functional enrichment, immune infiltration, somatic mutation, and nomogram-based survival prediction, were conducted alongside in vitro experiments. Additionally, drug sensitivity and Chinese medicine prediction analyses were performed to identify potential therapeutic agents for GBM.

**Results:** We detected a significant increase in global lactylation levels in GBM, which correlated with patient survival. We identified 227 differentially expressed HLMRGs from the intersection of 3,343 differentially expressed genes and 948 key module genes, indicating strong prognostic potential. Notably, genes such as SNCAIP, TMEM100, NLRP11, HOXC11, and HOXD10 were highly expressed in GBM. Functional analysis suggested that HLMRGs are involved primarily in pathways related to cytokine‒cytokine receptor interactions, cell cycle regulation, and cellular interactions, including microglial differentiation states. Further connections were established between HLMRGs and infiltrating immune cells, particularly type 1 T helper (Th1) cells, as well as mutations in genes such as PTEN. The potential therapeutic agents identified included ATRA and Can Sha.

**Conclusion:** The HLM-related gene risk prediction model shows substantial promise for improving patient management in GBM, providing crucial insights for clinical prognostic evaluations and immunotherapeutic approaches in GBM.

## 1. Introduction

Gliomas, arising from the neoplastic transformation of glial or neuronal progenitor cells within the central nervous system, represent approximately 32% of all intracranial neoplasms. Among these gliomas, malignant gliomas are the most prevalent and lethal brain tumors. According to the WHO classification system, gliomas are categorized into grades 1 through 4, with glioblastoma (GBM) corresponding to grade 4. GBM represents approximately 57% of all gliomas and 48% of primary malignant central nervous system tumors, with an incidence rate of 3.21 per 100,000 individuals 1-3. GBM is characterized by aggressive proliferation, invasion, angiogenesis, heterogeneity, and immunosuppression and remains a significant clinical challenge. In the face of advancements in therapeutic approaches, which encompass surgical interventions, radiation therapy, chemotherapy, and targeted treatments, the median survival for GBM patients remains dishearteningly short at just 15 months, with respective 2-year and 5-year survival rates of 26.5% and a mere 5.8%^1^, ^4^-^6^. Prognostic and predictive biomarkers play indispensable roles in assessing patient outcomes and guiding therapeutic strategies. Identifying novel prognostic genes is crucial for elucidating GBM pathogenesis, improving the prognosis management of patients, optimizing personalized treatment approaches, and facilitating the development of targeted therapies.

Glycolysis refers to the process in which glucose is broken down into pyruvate in the cytoplasm under anaerobic conditions and a small amount of ATP is produced, which belongs to a type of glucose metabolism 7, 8. Glycolysis is of great importance in organisms as it is the main pathway of glucose breakdown under hypoxic conditions and also provides rapid energy during strenuous exercise. Tumor cells adapt to the altered metabolic environment by switching between glycolysis and oxidative phosphorylation. Aerobic glycolysis means that the tumor consumes more glucose than the surrounding normal tissue, and glucose can be fermented to produce lactate, with the consequence of aerobic glycolysis being increased intracellular and extracellular lactate concentrations 9, 10. The increased demand for ATP metabolism in tumor cells leads to the occurrence of glycolysis, which is also the reason for the higher concentration of lactate in tumor cells than in non-tumor cells 9, 11. Lactate produced by glycolysis in tumor cells may bias tumor-associated macrophages toward an immunosuppressive phenotype, tumor progression is associated with the accumulation of lactate produced by aerobic glycolysis in the tumor microenvironment 12. A large number of studies have shown that lactate is produced by aerobic glycolysis under a variety of conditions, such as trauma, inflammation, infection, myocardial infarction, and tumor 13. This indicates that glycolysis and lactate have an important effect on our body.

Lactate, a byproduct of glycolysis under hypoxic conditions, plays a multifaceted role in tumor biology. Tumor cells predominantly rely on glycolysis to convert glucose to lactate, even in the presence of oxygen—a metabolic reprogramming known as the Warburg effect 14, 15. Lactate functions as an energy source, promotes angiogenesis, acts as a signalling molecule, and regulates immune responses 16, 17. Within glioblastoma multiforme, lactate has emerged as a pivotal activator of oxidative metabolism, enhancing tumor cell proliferation, longevity, and migratory potential 18, 19. Furthermore, lactate production inhibitors have reprogrammed the glucose metabolism of cancer stem cells, thereby alleviating the immunosuppression within the tumor microenvironment 20.

Histones are basic proteins in the chromatin of eukaryotic cells, which together with DNA form the nucleosome structure. Posttranslational chemical modifications of histones represent a key mechanism of epigenetic regulation and play a pivotal role in gene expression 21. Among these modifications, histone lactylation modification (HLM), characterized by the addition of a lactoyl group derived from lactoyl-CoA to lysine residues, represents a significant advancement in the study of lactate biology. HLMs have been implicated in various pathological processes, including tumor progression, inflammation, sepsis, pulmonary hypertension, Alzheimer's disease, and renal fibrosis 22-27. Warburg effect induced histone lactylation drives NF-κB-associated LINC01127 expression through MAP4K4/JNK/NF-κB axis to promote self-renewal of GBM cells 28. Furthermore, increased histone H3K9 lactylation has been observed in recurrent GBM, with chronic temozolomide exposure contributing to elevated H3K9 lactylation levels 29. These findings suggest a critical role for HLM in GBM pathogenesis; however, the precise mechanisms of HLM-related genes (HLMRGs) in GBM remain unexplored.

Single-cell RNA sequencing (scRNA-seq) is a powerful high-throughput technology that enables cell-specific transcriptomic analysis at single-cell resolution. This technique has been extensively employed in studies of embryonic development and tumor biology. By elucidating the gene structure and expression status of individual cells, scRNA-seq distinguishes functionally normal cells from cancer cells across different stages of tumor development, thereby advancing molecular-level investigations 30, 31. Studies have reported that scRNA-seq analysis of different GBM cells revealed inherent differences in the expression of different transcriptional programs related to oncogenic signaling, proliferation, immune response, and hypoxia 32. This technology has proven invaluable in studying tumor heterogeneity, including understanding tumor evolution, uncovering chemotherapy resistance mechanisms, and informing novel therapeutic strategies 33.

By leveraging transcriptome and scRNA-seq data from publicly available databases, this study identified HLM-related prognostic genes in GBM through bioinformatics analyses and developed a prognostic risk model to predict patient survival. Additionally, the biological functions and molecular mechanisms of the identified prognostic genes were systematically explored. These findings provide new perspectives for clinical prognosis prediction and targeted drug development in GBM.

## 2. Methods

### 2.1 Clinical samples

The tumor tissues used for this study were obtained from patients with gliomas who underwent surgical resection at the Department of Neurosurgery, Affiliated Hospital of Southwest Medical University. Ethical approval was granted by the ethics committee of the Affiliated Hospital of Southwest Medical University, and all participants provided written informed consent.

### 2.2 Data sources

The TCGA-GBM dataset, comprising 169 GBM tissue samples from 168 patients (167 samples from 166 patients with survival information) and 5 normal tissue samples from 5 individuals, was retrieved from The Cancer Genome Atlas (TCGA) database (https://portal.gdc.cancer.gov/) and utilized as the training set. Additionally, the CGGA-GBM dataset, containing 693 GBM tissue samples from 693 patients (413 samples from 413 patients with survival information), was sourced from the Chinese Glioma Genome Atlas (CGGA) database (http://www.cgga.org.cn/) and served as the validation set. The scRNA-seq dataset GSE162631 (GPL24676), which included 4 GBM and 4 normal tissue samples, was acquired from the Gene Expression Omnibus (GEO) database (https://www.ncbi.nlm.nih.gov/geo/). A total of 14 HLMRGs were identified from the literature 34-36.

### 2.3 Association analysis between the global HLM level and GBM

To assess the associations between HLM levels and the survival of patients with GBM, the 14 HLMRGs in the TCGA-GBM dataset were subjected to single-sample gene set enrichment analysis (ssGSEA) via the GSVA package (v1.42.0) 37. The ssGSEA scores of the HLMRGs were compared between GBM and normal samples via the Wilcoxon rank-sum test, with P < 0.05 indicating statistical significance.

GBM samples were classified into high- and low-scoring groups on the basis of the optimal cut-off value of the ssGSEA score. Kaplan‒Meier (KM) survival curves for these groups were generated via the survival package (v3.5--5) 38, and survival differences were evaluated via the log-rank test, with P < 0.05 indicating statistical significance.

### 2.4 Functional enrichment and protein‒protein interaction (PPI) analyses of HLMRGs in GBM

To identify differentially expressed genes (DEGs) in the TCGA-GBM dataset, differential expression analysis was conducted via the DESeq2 package (v1.40.2) 39 under the criteria of a |log2-fold change (FC)| > 2 and adjusted P < 0.05. Visualization of the DEGs was performed via the ggplot2 package (v3.5.0) 40 for generating volcano plots and the ComplexHeatmap package (v2.12.1) 41 for creating heatmaps.

After unqualified genes and samples were filtered and the data were clustered via the GoodSamplesGenes function, weighted gene coexpression network analysis (WGCNA) was conducted within the TCGA-GBM dataset. The pickSoftThreshold function was used to determine an optimal threshold, ensuring that gene interactions conformed to a scale-free network. Genes were subsequently grouped into modules through hierarchical clustering, with parameters set to minModuleSize = 30, mergeCutHeight = 0.3, and verbose = 5. The Pearson correlation coefficient was applied to evaluate the relationships between module eigengene (ME) scores and the ssGSEA scores of the HLMRGs via the WGCNA package (v1.71) 42. The genes in the module whose ME scores had the highest correlation with the ssGSEA score of the HLMRGs were defined as key module genes.

To identify candidate genes, the VennDiagram package (v1.7.1)^43^ was employed to find the intersection between DEGs and key module genes, representing genes closely associated with HLM in GBM. Functional enrichment analyses of these candidate genes were performed via the clusterProfiler package (v4.2.2) 44 for Gene Ontology (GO) and Kyoto Encyclopedia of Genes and Genomes (KEGG) pathway analyses, with statistical significance set at P < 0.05. To explore protein-level interactions among the candidate genes, the Search Tool for the Retrieval of Interacting Genes/Proteins (STRING) database (https://cn.string-db.org/) was utilized to construct a protein‒protein interaction (PPI) network, with a confidence score threshold > 0.9. High-quality interactions, defined as those with at least 30 connections and coexpressions and experimentally determined interaction scores > 0.6, were visualized via Cytoscape software (v3.1.1)^45^.

### 2.5 Construction and validation of a risk model based on HLMRGs

To evaluate the potential prognostic value of candidate genes in predicting overall survival (OS) in patients with GBM, univariate Cox regression analysis was performed on the TCGA-GBM dataset, and genes with hazard ratios (HR ≠ 1) and P < 0.05 were selected. The survival package (v3.5-3) 38 was used to conduct a proportional hazards (PH) assumption test, retaining genes with P > 0.05. These candidate prognostic genes were visualized via the forestplot package (v2.0.1) 46. Subsequently, least absolute shrinkage and selection operator (LASSO) regression analysis was conducted via the glmnet package (v4.1--4) 46 to identify key prognostic genes. Differential expression of the prognostic genes between GBM and normal samples in the TCGA-GBM dataset was analysed via the Wilcoxon rank-sum test (P < 0.05) to validate their expression profiles.

A risk model was constructed on the basis of the identified prognostic genes via the following formula:

Risk score
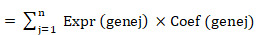


In this formula, "coef" represents the LASSO regression coefficient, and "expr" denotes the expression level of each prognostic gene.

Patients with GBM were stratified into high- and low-risk groups on the basis of the optimal cut-off values of risk scores. The survminer package (v0.4.9) 47 and ggrisk package (v1.3) 48 were used to visualize the risk score distribution, survival state distribution, and gene expression heatmap across the risk groups. KM survival curves comparing high- and low-risk groups were generated via the survminer and survival packages, with survival differences assessed via the log-rank test (P < 0.05). To assess the model's predictive performance, receiver operating characteristic (ROC) curves were plotted at 3, 5, and 7 years via the pROC package (v1.18.0) 49, and the area under the curve (AUC) was calculated. To validate the accuracy and generalizability of the HLMRGS model, the same analysis was performed within the CGGA-GBM dataset.

### 2.6 Gene set enrichment analysis

To elucidate the pathways and biological mechanisms associated with HLMRGS, differential expression analysis between the high- and low-risk groups within the TCGA-GBM dataset was performed via the DESeq2 package (v 1.40.2). Genes were ranked on the basis of their log2-fold change (log2 FC) in descending order, and gene set enrichment analysis (GSEA) was conducted via the clusterProfiler package (v4.2.2) with an adjusted P < 0.05. The reference gene set "c2.cp.kegg.v7.2.symbols.gmt" was sourced from the msigdbr package (v7.5.1) 50.

Additionally, GSEA was applied to prognostic genes within the TCGA-GBM dataset to investigate their functional roles and pathway differences. Using the KEGG pathway reference gene set "c2.cp.kegg.v7.2.symbols.gmt," Spearman correlation coefficients between prognostic genes and other genes were calculated with the cor function, applying thresholds of |cor| > 0.3 and P < 0.05. The resulting correlation coefficients were ranked in descending order, and individual GSEA analyses for each prognostic gene were performed via the clusterProfiler package, with an adjusted P < 0.05 as the significance criterion.

### 2.7 scRNA-seq data processing and expression pattern analysis of prognostic genes

To further examine the expression patterns of prognostic genes at the single-cell level, analyses were conducted via the scRNA-seq dataset.

The percentageFeatureSet function in the Seurat package (v5.1.0) 51 was applied for the initial filtering of the scRNA-seq data. Genes expressed in more than three cells were retained, while cells containing between 200 and 8,000 genes and gene counts ranging from 200--30,000 were included. Additionally, mitochondrial gene percentages were calculated, and cells whose mitochondrial gene content was less than 10% were selected. The data were standardized via the NormalizeData function (LogNormalize, scale.factor = 10,000), and the top 2,000 genes with the largest variance were identified via the FindVariableFeatures function. Further normalization was conducted with the ScaleData function. Principal component analysis (PCA) was performed via the RunPCA, ElbowPlot, and JackStraw functions, and principal components were identified for subsequent analyses (P < 0.05).

Following PCA, cell annotation was performed via the FindAllMarkers function (|log2 FC| > 0.5, P < 0.05) on the basis of marker genes from the literature 52. Visualization of the annotation results was achieved through uniform manifold approximation and projection (UMAP) and bubble plots. Cell type proportions were calculated for the GBM and normal groups, and the cell type showing the greatest difference and highest proportion in the GBM group was identified as the key cell type.

Cell‒cell communication analysis was then conducted to investigate intercellular interactions between key cell types and other annotated cell types in both the GBM and normal groups via the CellChat package (v1.5.0) 53 (P < 0.05). To explore potential ligand‒receptor interactions, the CellPhoneDB database (v2.0) (https://www.cellphonedb.org/) was used for further analysis.

For a deeper understanding of the expression dynamics of prognostic genes, cell trajectory analysis was performed on the key cell types via the Monocle package (v2.26.0) 54. This analysis simulated the differentiation trajectory of key cell types and assessed the expression trends of prognostic genes across different developmental stages.

### 2.8 Immune microenvironment analysis

Within the TCGA-GBM dataset, differences in the immune microenvironment between the HLMRGS groups were analysed. The relative proportions of 22 types of immune-infiltrating cells in the high- and low-risk groups, stratified by ssGSEA scores, were assessed and visualized via a stacked histogram. Immune cells with significant differences between groups were identified via the Wilcoxon rank-sum test (P < 0.05). Spearman correlation analysis was subsequently performed between these immune cells and prognostic genes via the psych package (v2.2.9) 55, and the results were visualized via the pheatmap package (v1.0.12) 41.

Somatic mutation data for patients with GBM were retrieved from the TCGA-GBM dataset. The maftools package (v2.20.0) (https://github.com/poisonalien/maftools) was utilized to analyse and visualize somatic mutation profiles for the high- and low-risk groups, providing insights into mutation patterns and their potential implications.

### 2.9 Independent prognostic analysis and nomogram establishment

To identify independent prognostic factors and evaluate the clinical applicability of the HLMRGS, age, sex, and risk score were subjected to univariate and multivariate Cox regression analyses (HR ≠ 1, P < 0.05). Factors that passed the proportional hazards (PH) assumption test (P > 0.05) were considered independent predictors. A nomogram model was constructed via the rms package (v6.7-0) 56 to predict survival probabilities at 3, 5, and 7 years. The model's predictive accuracy was evaluated by plotting ROC curves via the pROC package (v1.18.0), with the AUC calculated to quantify performance.

Independent prognostic analysis was conducted on the CGGA-GBM dataset to further validate the findings. Factors such as chemotherapy status, histology, O6-methylguanine-DNA-methyltransferase gene promoter (MGMTp), polygenic risk score (PRS), age, and sex were evaluated through univariate and multivariate Cox analyses (HR ≠ 1, P < 0.05) and the PH assumption test (P > 0.05). A nomogram model was then established to predict survival probabilities at 1, 2, and 3 years. Calibration curves, decision curve analysis (DCA), and receiver operating characteristic (ROC) curves were generated to assess the model's accuracy and clinical utility. These visualizations were produced via the rms package (v6.7-0) for calibration curves, the ggDCA package (v1.2) 57 for DCA curves, and the pROC package (v1.18.0) for ROC curves.

### 2.10 Drug sensitivity analysis and Chinese medicine prediction

To identify potential drugs for patients with GBM exhibiting high HLMRGS, 50% inhibitory concentration (IC50) values for 138 chemotherapy drugs from the Genomics of Drug Sensitivity in Cancer (GDSC) database (https://www.cancerrxgene.org/) were calculated for GBM samples in the TCGA-GBM dataset via the pRRophetic package (v0.5) 58. Differences in the IC50 values between the high- and low-risk groups were evaluated via the Wilcoxon rank-sum test (P < 0.05). The top 10 drugs with the lowest p values were visualized via the ggplot2 package (v3.5.0).

To predict Chinese medicines with potential therapeutic effects for patients with GBM, prognostic genes were first analysed via GO enrichment analysis via the clusterProfiler package (v4.2.2), with P < 0.05 as the significance threshold. The identified prognostic genes and biological processes (BP) related to immune infiltration were then input into the Coremine Medical database (http://www.coremine.com/medical/) to identify related Chinese medicines (P < 0.05). The network linking prognostic genes, immune infiltration-related BPs, and Chinese medicines was visualized via Cytoscape software (v3.1.1).

### 2.11 Cell culture and Western blotting

For in vitro experiments, T98G, U87MG, and U251MG cells were obtained from Sichuan Bio Biotech Co., Ltd. (Chengdu, China), and HA1800 cells were obtained from Guangzhou Jennio Biotech Co., Ltd. (Guangzhou, China). The cells were cultured in DMEM (Gibco, Shanghai, China) supplemented with 10% fetal bovine serum (Vazyme, Nanjing, China) and 1% penicillin‒streptomycin (Solarbio, Beijing, China) at 37°C in a 5% CO_2_ incubator.

For Western blot analysis, total protein was extracted via RIPA lysis buffer (P10013B; Beyotime, Shanghai, China) supplemented with a protease and phosphatase inhibitor cocktail (P1045; Beyotime, Shanghai, China) on ice and quantified via a BCA protein assay kit (P0011; Beyotime, Shanghai, China). Equal amounts of protein were resolved by SDS‒PAGE and transferred onto PVDF membranes (ISEQ00010, Merck Millipore, USA). The membranes were blocked with 5% skim milk for 1 hour at room temperature, followed by overnight incubation with the following primary antibodies at 4°C: Kla (PTM-1401RM, PTMBIO, Hangzhou, China), HOXC11 (P12672, ProMab, Shanghai, China), HOXD10 (P05871, ProMab, Shanghai, China), SNCAIP (P34200, ProMab, Shanghai, China), TMEM100 (P30991, ProMab, Shanghai, China), and NLRP11 (PAB37681, Bioswamp, Wuhan, China). The membranes were then incubated with secondary antibodies (SA00001-1/SA00001-2; Proteintech, Wuhan, China) for 1 hour at room temperature. Protein bands were visualized via the SuperPico ECL Chemiluminescence Kit (E422-02, Vazyme, Nanjing, China) and standardized against histone H3 (MB9211S, Abmart, Shanghai, China) and β-actin (66009-1-Ig, Proteintech, Wuhan, China). Band intensities were analysed via ImageJ software.

### 2.12 Immunohistochemistry

Paraffin-embedded tissue samples were sectioned, deparaffinized, and subjected to antigen retrieval. Endogenous peroxidase activity was blocked, followed by serum blocking. The sections were incubated overnight at 4°C with the primary antibody Kla (1:100, PTM-1401RM, PTMBIO, Hangzhou, China) and then with the secondary antibody at room temperature for 50 minutes. Detection was carried out via DAB staining, and the nuclei were counterstained. The slides were subsequently dehydrated, sealed, and visualized under a microscope for interpretation of the results.

### 2.13 Lactate measurement

Cells were harvested by trypsin digestion, washed with PBS, and subjected to ultrasonic disruption on ice. After centrifugation, the supernatant was collected. Following the protocol of the lactate assay kit, samples were reacted and absorbance was measured using a microplate reader. Simultaneously, protein concentration was determined via the BCA method to normalize lactate content in the final calculation.

### 2.14 Statistical analysis

Statistical analyses were conducted via GraphPad Prism 9, whereas bioinformatics analyses were performed via R (v4.2.2). Group comparisons were carried out via the Wilcoxon rank-sum test, Student's t test, or one-way ANOVA, as appropriate. The data are presented as the means ± standard deviations, with P < 0.05 considered statistically significant.

## 3. Results

### 3.1 Global HLM levels are increased in GBM patients and associated with survival

**Figure 1:**
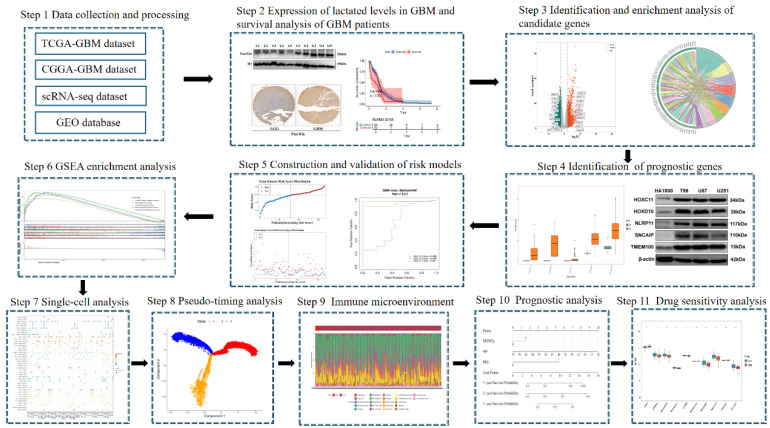
Flow chart of this study.

**Figure 2:**
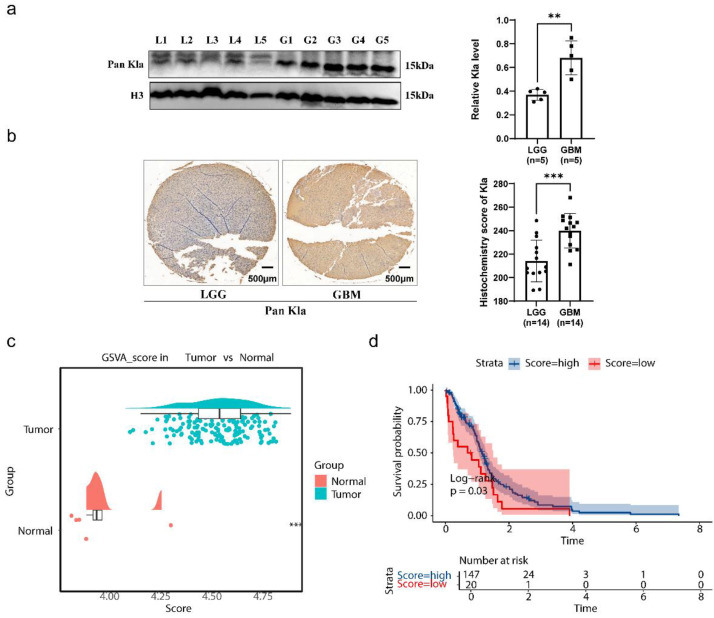
** Validation of clinical samples for gliomas.** (a) Results of western blot validation. (b) Results of immunohistochemical staining. (c) Results of ssGSEA. (d) Results of the KM survival analysis.

This study aimed to evaluate the clinical significance and potential value of HLM in GBM (**Figure 1**). Western blot analysis of glioma clinical samples revealed elevated global lactylation levels in GBM compared with low-grade glioma (LGG) (**Figure 2a**). Immunohistochemical staining further validated this observation, revealing increased global lactylation levels in GBM tissue samples relative to LGG samples (**Figure 2b**). ssGSEA revealed that the ssGSEA scores of the HLMRGs were significantly greater in the GBM samples than in the normal control samples (P < 0.05) (**Figure 2c**).

Using the optimal cut-off value for the ssGSEA scores of the HLMRGs (4.350889), the GBM samples were stratified into high- and low-scoring groups. K‒M survival analysis revealed significant differences in survival between the two groups (P = 0.03), with patients in the low-score group exhibiting poorer survival probabilities. These findings suggest an association between global HLM levels and the survival and prognosis of patients with GBM (**Figure 2d**). In parallel with these findings, we have incorporated comprehensive lactate quantification data comparing normal astrocytes with GBM cell lines in Supplementary Figure S7. Quantitative analysis revealed that GBM cell lines demonstrated markedly elevated lactate levels compared to HA1800 astrocytes (p < 0.0001). The demographic and clinical characteristics of the study cohort are comprehensively summarized in Table S1.

### 3.2 Related functional pathways and complex PPI network of HLMRGs in GBM

**Figure 3:**
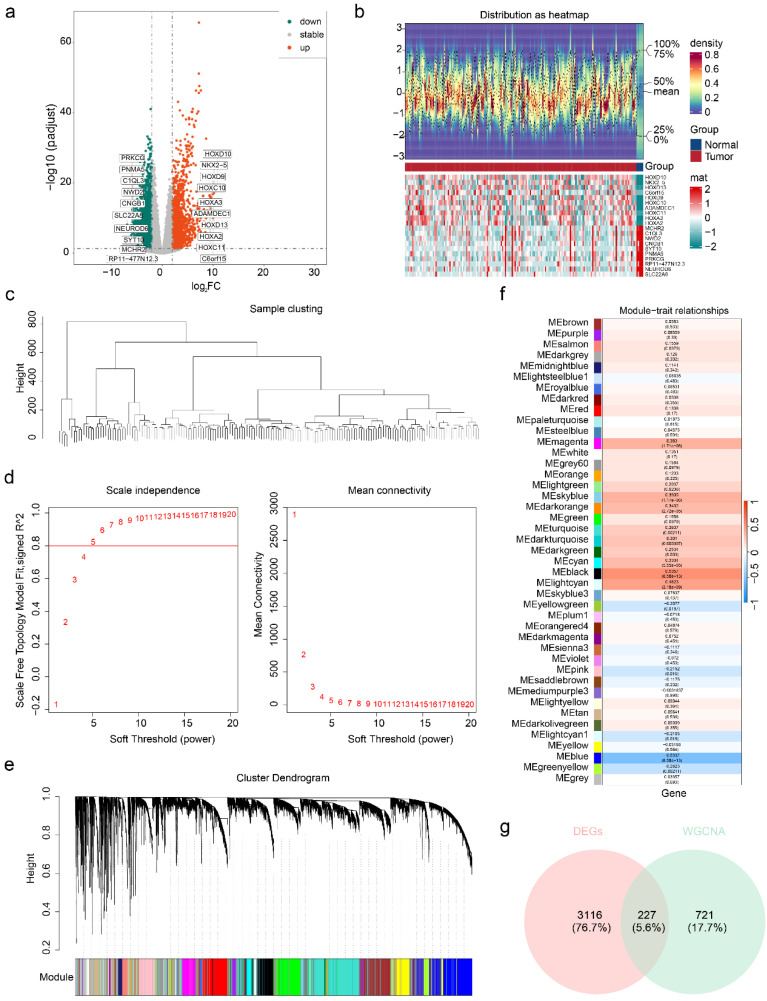
**Identification of candidate genes.** (a) Volcano map of differentially expressed genes. The green dots indicate genes whose expression was differentially downregulated, the red dots indicate genes whose expression was differentially upregulated, the gray dots indicate genes whose expression was not significantly different, and the names of the genes whose expression was differentially upregulated and downregulated in the top ten multiplicities of difference are indicated. (b) Heatmap of differentially expressed genes. (c) Results of sample clustering. (d) Screening of soft thresholds. (e) Dendrogram of gene clusters. (f) Heatmap of the relationships between the HLMRG scores and color modules are shown on the left, and the color bands on the right represent correlations. In the middle heatmap, darker colours indicate greater correlations, blue represents negative correlations, red represents positive correlations, and the numbers in each cell indicate correlations and significance (in parentheses). (g) Venn diagram used to identify candidate genes.

A total of 3,343 DEGs were identified between the GBM and normal groups, including 1,577 upregulated genes and 1,766 downregulated genes. A volcano plot highlighted the most significantly upregulated and downregulated genes, with the top 10 genes labelled (**Figure 3a**). The expression patterns of these DEGs were visualized via a heatmap (**Figure 3b**).

Following sample clustering and confirmation of data quality (**Figure 3c**), WGCNA was performed. An optimal soft threshold of 5 was selected on the basis of the scale-free topology fit index (signed R2 = 0.8) and mean connectivity. This analysis grouped genes into 42 coexpression modules (**Figure 3d-e**). Among these, the MEblack module, containing 948 genes, exhibited the strongest correlation with the ssGSEA scores of the HLMRGs (cor = 0.5357015, P < 0.001) and was identified as the key module (**Figure 3f**).

From the intersection of the DEGs and key module genes, 227 candidate genes associated with HLM in GBM were identified (**Figure 3g**). GO enrichment analysis revealed significant enrichment in 403 entries, including 318 BP, 57 CC, and 28 MF terms. Key enriched processes, such as chromosome segregation, nuclear division, and organelle fission, were visualized (**Figure S1a**). KEGG pathway analysis revealed 25 significantly enriched pathways, including those related to the cell cycle, oocyte meiosis, and cellular senescence, with the top 10 pathways highlighted (**Figure S1b**). These findings suggest that candidate genes are involved primarily in critical cellular processes and pathways, shedding light on the functional roles of HLMRGs in GBM. PPI network analysis further revealed 153 proteins and 1,287 interaction pairs, highlighting the complex interactions among proteins encoded by the candidate genes. These findings underscore the potential importance of HLMRGs in GBM cellular functions and progression (**Figure S1c**).

### 3.3 HLMRGs demonstrated strong predictive power for GBM prognosis

**Figure 4:**
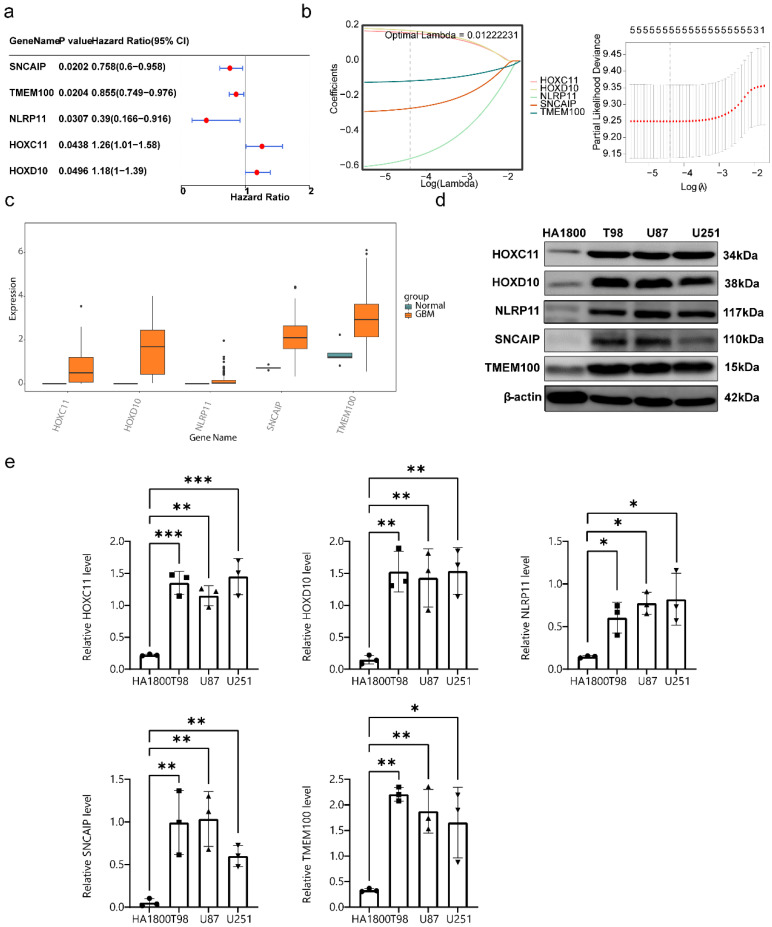
** Screening for prognostic genes.** (a) Results of univariate Cox analysis. (b) Results of LASSO regression analysis. (c) Expression of prognostic genes in the GBM and control groups. (d-e) Protein expression level analysis of 5 prognostic genes.

Univariate Cox analysis identified SNCAIP, TMEM100, NLRP11, HOXC11, and HOXD10 as candidate prognostic genes (**Figure 4a**). Among these, SNCAIP, TMEM100, and NLRP11 were associated with better prognoses (HR < 1), suggesting their potential inhibitory effects on GBM progression. In contrast, HOXC11 and HOXD10 were linked to worse prognoses (HR > 1), indicating their possible roles in promoting GBM progression. Additionally, the PH assumption test confirmed that these genes satisfied the PH assumption (P > 0.05) (**Table S2**). LASSO regression analysis further validated these five genes as key prognostic markers (lambda min = 0.01222231) (**Figure 4b**). All five genes presented significantly higher expression levels in GBM samples than in normal control samples, highlighting their relevance to GBM progression and their potential as therapeutic targets (**Figure 4c**). The protein expression levels of these genes were verified in normal human astrocytes and three GBM cell lines (T98G, U87MG, and U251MG) through Western blot analysis. The results demonstrated that the protein levels of the five prognostic genes were significantly elevated in GBM cell lines compared with normal astrocytes, which aligns with the gene expression analysis results (**Figure 4d-e**). In parallel with these findings, we have supplemented the data on Kla protein levels in normal astrocytes and different GBM cell lines, as shown in Figure S8. The results revealed that compared to HA1800 cells, the expression levels of Kla in GBM cell lines were significantly higher, with statistically significant differences. This trend aligns with the detection results of prognostic genes in GBM cell lines, indicating that there is indeed a correlation between prognostic genes and lactylation.

**Figure 5:**
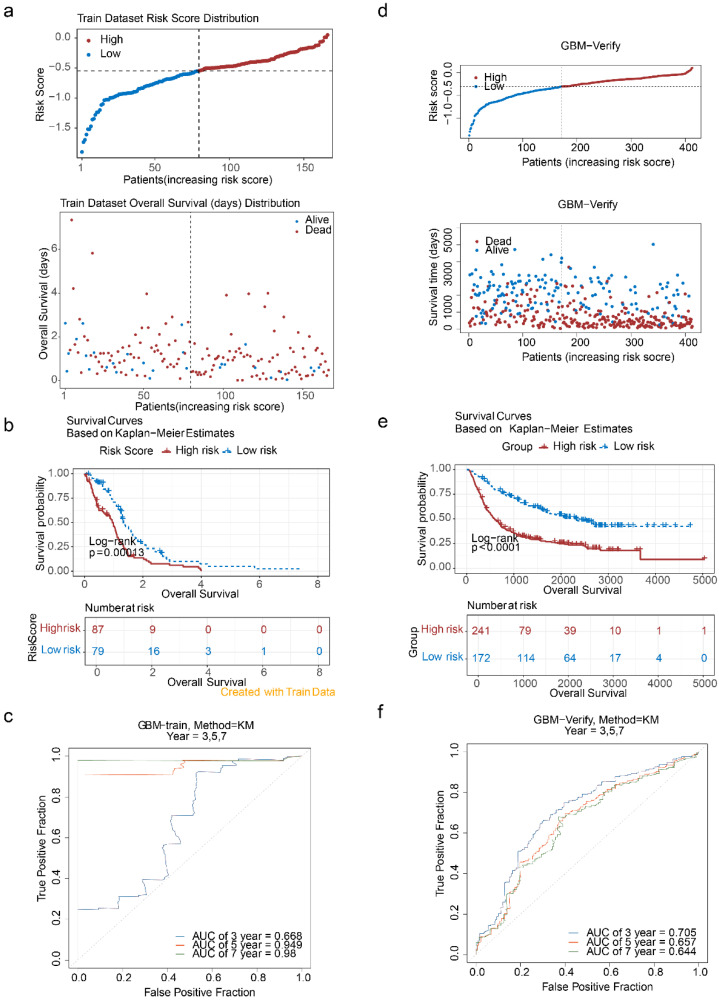
** Construction of prognostic models.** (a-1) Risk score distribution of GBM patients. (a-2) Survival state distribution of GBM patients. (b) K‒M curves for patients in the high- and low-risk groups. (c) ROC curves for patients at 3, 5 and 7 years. (d-1) Risk score distributions of the high- and low-risk groups. (d-2) Survival distribution of the high- and low-risk groups. (e) Distribution of the KM survival curve. (f) Distribution of the ROC curve.

On the basis of the identified prognostic genes, a risk model named the HLMRGS model was constructed via the following formula: risk score = (-0.2698277) × SNCAIP expression level + (-0.1142342) × TMEM100 expression level + (-0.5557958) × NLRP11 expression level + (0.1585500) × HOXC11 expression level + (0.1718749) × HOXD10 expression. Using this model, 166 patients with GBM from the TCGA-GBM dataset were stratified into high- and low-risk groups (87:79) on the basis of the optimal cut-off value (-0.5511703). Risk score distribution and survival state analyses demonstrated that higher risk scores were correlated with increased mortality in patients with GBM (**Figure 5a**). KM survival analysis revealed that patients in the low-risk group had significantly better survival probabilities (P < 0.001) (**Figure 5b**). The AUCs for 3-, 5-, and 7-year survival exceeded 0.6, reflecting the strong predictive performance of the model (**Figure 5c**).

To validate the model, it was applied to the CGGA-GBM dataset, where 413 patients with GBM were similarly divided into high- and low-risk groups (241:172) on the basis of an optimal cut-off value (-0.3031568). The distributions of risk scores and survival states (**Figure 5d**), KM survival curves (**Figure 5e**), and ROC curves (AUC exceeding 0.6) (**Figure 5f**) were consistent with the results from the TCGA-GBM dataset. These findings underscore the robustness and generalizability of the HLMRGS model, demonstrating its efficacy in stratifying patient risk and predicting survival outcomes across independent datasets. The HLMRGS model provides a promising tool for personalized prognostic assessment in GBM clinical practice, offering valuable insights for tailoring patient management and improving outcome predictions. We used the "ssGSEA" algorithm in the R package "GSVA" to calculate HLMRGs scores with WHO grades in the CGGA dataset, and used Wilcoxon rank sum test (p<0.05) to analyze the differences in HLMRGs scores among different WHO grades (**Figure S9**). The results showed that HLMRGs scores were significantly different between WHO IV grade and WHO II, WHO III grade samples.

### 3.4 Potential mechanisms associated with HLMRGS and prognostic genes

Pathway enrichment analysis via GSEA within the TCGA-GBM dataset revealed that DEGs between the high- and low-risk HLMRGS groups were significantly associated with pathways such as cytokine‒cytokine receptor interactions, hematopoietic cell lineages, and chemokine signalling (**Figure S2a**). These results suggest the multifaceted roles of differentially expressed genes in GBM pathogenesis. Additionally, pathways associated with the identified prognostic genes were examined, highlighting the five pathways most significantly associated with each gene. SNCAIP was enriched in 55 pathways, including allograft rejection (**Figure S2b**). TMEM100 and NLRP11 are linked to pathways such as the cell cycle (51 and 14 pathways, respectively) (**Figure S2c-d**). HOXC11 was associated with 54 pathways, including the calcium signalling pathway (**Figure S2e**), and HOXD10 was enriched in 33 pathways, such as antigen processing and presentation (**Figure S2f**). Notably, pathways such as the cell cycle and spliceosome were enriched across multiple prognostic genes, emphasizing their potential roles in GBM progression. These findings underscore the importance of further exploration of these pathways to elucidate how HLM levels influence GBM progression. Therapeutic strategies targeting prognostic genes or these pathways may offer innovative approaches for GBM treatment.

**Figure 6:**
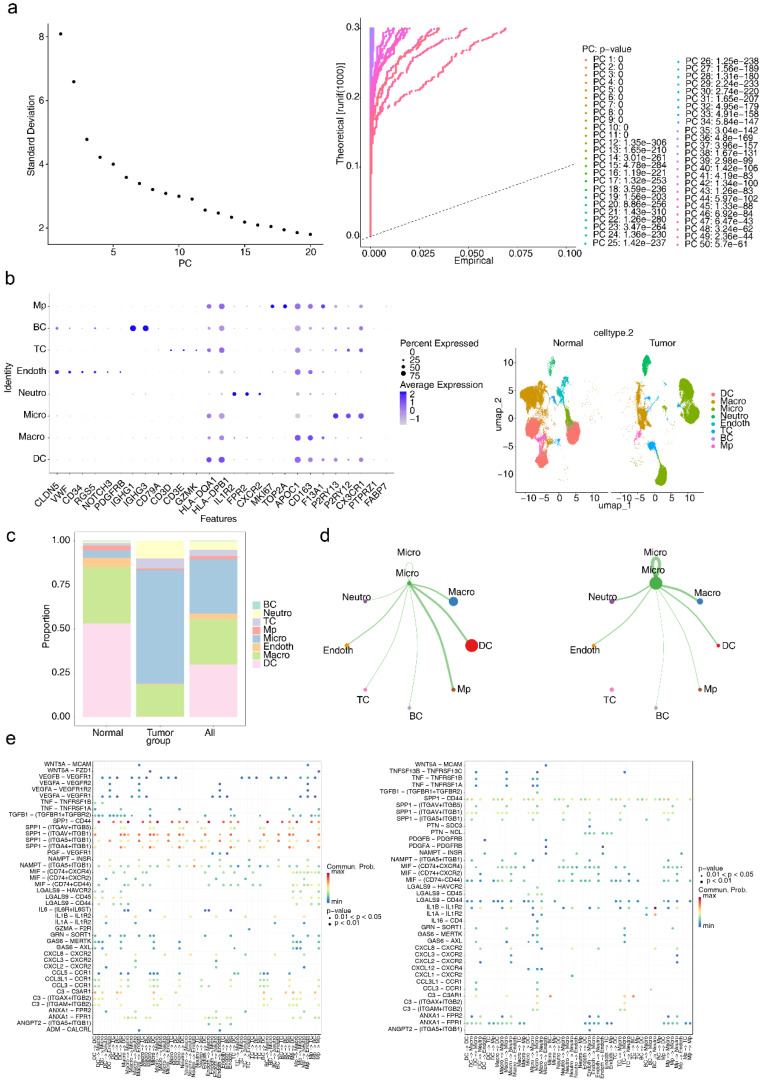
** Analysis of cellular communication.** (a-1) Inflection plot for PCA. (a-2) The top 20 principal components of the PCA. (b-1) Bubble diagram of marker gene expression in different cell types. (b-2) Visualization of cell clusters after cell annotation. (c) Proportion of annotated cells in the GBM and normal groups. (d) Communication network between Micro and different cells. (e) Specific receptor‒ligand interactions between different cell types.

At the single-cell level, scRNA-seq data were processed to investigate the underlying mechanisms involved. Initial filtering and quality control metrics, including nFeature RNA, nCount RNA, and percent.mt, are presented in **Figure S3**. After filtering, the integrated dataset comprised 95,428 cells and 24,983 genes. The degree of variation in these genes, with the top 10 most variable genes labelled, is shown in **Figure S4.** PCA confirmed that the cells from all eight samples were well integrated without outliers (**Figure S5**), validating the suitability of the dataset for further analysis. On the basis of the inflection point plot and PCA replacement test, the top 20 principal components were retained (**Figure 6a**). The cells were subsequently clustered and annotated into eight subtypes via marker genes, including dendritic cells (DCs), macrophages (Macro), microglia (Micro), neutrophils (Neutro), endothelial cells (Endoth), T cells (TCs), B cells (BCs), and proliferating macrophages (MPs) (**Figure 6b**). The proportions of these cell types in the GBM and normal groups were compared, revealing that DCs were most abundant in the normal group, whereas microglia were present in the highest proportion in the GBM group (**Figure 6c**). Given the established association between microglia and GBM progression, microglia were identified as the key cell type for further analysis.

Intercellular interactions between Micro and other cell types were further analysed. In the normal group, Micro exhibited interactions with all other cell types, with the strongest interactions observed between Micro and DCs and MPs. Conversely, in the GBM group, no interactions were detected between Micro and TCs, whereas interactions with Macro and Neutro were the strongest (**Figure 6d**). These findings highlight the distinct roles of various cell types in the intercellular communication networks of the GBM and normal groups. Ligand‒receptor interactions were also examined, with notable differences identified. The SPP1‒CD44 axis is strongly implicated in numerous interactions, such as Micro-TCs, in the normal group but plays a diminished role in GBM, suggesting that interactions such as Micro-TCs are highly influenced by the SPP1‒CD44 ligand‒receptor pair and are significantly affected in the GBM microenvironment (**Figure 6e**).

The differentiation trajectories of Micro plants were analysed to illustrate their dynamic behavior over time, with darker colors in the graph indicating earlier cell differentiation (**Figure S6a**). The trajectory revealed three distinct differentiation states of Micro (**Figure S6b**). In the normal group, subpopulation 2 was the dominant state, whereas subpopulations 1 and 3 were predominant in the GBM group (**Figure S6c**). Additionally, a greater proportion of cells was observed at the terminal differentiation stages in the GBM group than in the normal group, indicating increased heterogeneity of Micro in the GBM group (**Figure S6d**). These findings suggest that Micro plays a pivotal role in GBM progression. Notably, no significant differences were found in the expression patterns of prognostic genes within Micro across distinct differentiation states (**Figure S6e**). These findings indicate that changes in the proportions of microsubpopulations, rather than the expression of prognostic genes, might be critical in GBM progression. These results suggest that the role of HLM in GBM progression and prognosis may involve additional cellular or molecular mechanisms beyond the direct expression patterns of HLMRGs in key cell types.

### 3.5 The role of HLMRGs in the immune microenvironment

**Figure 7:**
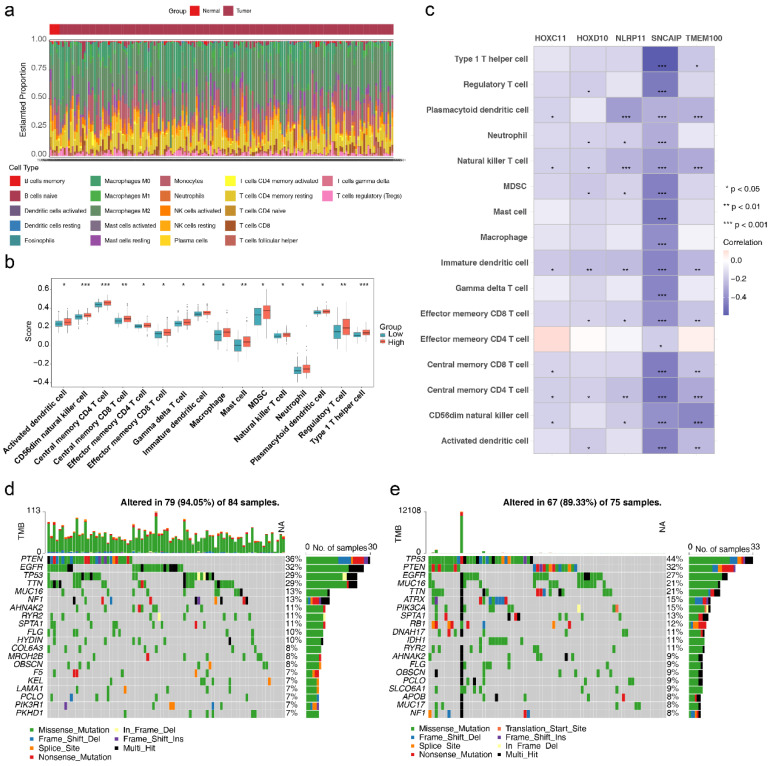
** Results of immune infiltration analysis.** (a) Relative percentages of 22 immune cell types. (b) Infiltration scores for immune cell types. (c) Correlations between differential immune cell types and prognostic genes. (d) Somatic mutations in high-risk groups. (e) Somatic mutations in low-risk groups.

The relative percentages of 22 immune cell types differed significantly between the high- and low-risk groups, as determined by HLMRGS (**Figure 7a**). Among these, 16 immune cell types, including activated dendritic cells, had significantly higher infiltration scores in the high-risk group (**Figure 7b**). Correlation analysis between these differential immune cell types and prognostic genes revealed inverse relationships (P < 0.05), with the strongest negative correlation observed between SNCAIP and type 1 T helper (Th1) cells (|cor| > 0.3, P < 0.05) (**Figure 7c**). These findings suggest that the immune microenvironment is significantly altered between risk groups, underscoring the role of HLM in modulating immune responses. Furthermore, the identified prognostic genes offer potential targets for personalized therapeutic strategies in GBM.

The somatic mutation profiles of the two risk groups were also examined (**Figure 7d-e**). Missense mutations dominated in both groups. PTEN mutations were present in 36% of the high-risk group, whereas TP53 mutations were observed in 44% of the low-risk group. These results imply that HLM levels may influence tumor mutation profiles, providing insights into the interplay between HLM and genomic alterations. These findings offer potential avenues for personalized treatment strategies and refined prognostic assessments in GBM.

### 3.6 A nomogram integrating the HLMRGS and clinical characteristics was established for accurate prediction

**Figure 8:**
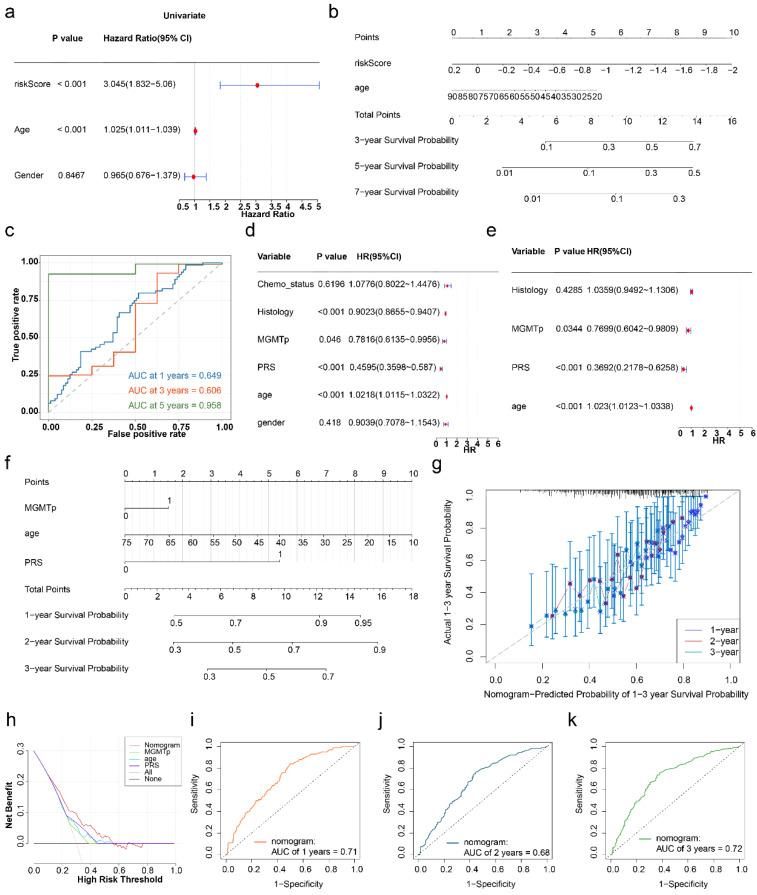
** Construction of the nomogram.** (a) Independent prognostic analysis of the TCGA-GBM dataset. (b) Construction of a nomogram in the TCGA-GBM dataset. (c) ROC curves for the nomogram. (d-e) Independent prognostic analysis of the CGGA-GBM dataset. (f) Construction of a nomogram in the CGGA-GBM dataset. (g) Calibration curve for the nomogram. (h) DCA curves for the nomogram. (i-k) ROC curves for the nomogram in the CGGA-GBM dataset.

Cox regression analysis of the TCGA-GBM dataset revealed that risk score and age were independent prognostic factors (P < 0.001) (**Figure 8a**), both of which satisfied the PH assumption (P > 0.05) (**Table S3**). A nomogram was constructed, which demonstrated that higher total points corresponded to improved survival probabilities (**Figure 8b**). The model's predictive accuracy was robust, with AUCs for 3-, 5-, and 7-year survival exceeding 0.6, validating its effectiveness as a clinical tool for GBM prognosis (**Figure 8c**).

In the CGGA-GBM dataset, age, PRS, and MGMTp were identified as independent prognostic factors (P < 0.05) (**Figure 8d-e**), and the PH assumption test confirmed their validity (P > 0.05) (**Table S4**). A corresponding nomogram was established (**Figure 8f**), with a calibration curve slope close to 1, indicating excellent agreement between the predicted and observed survival probabilities. DCA demonstrated a greater net benefit for the nomogram than for the individual genes did (**Figure 8g-h**). The model's AUCs for 1-, 2-, and 3-year survival exceeded 0.6, confirming its superior predictive ability (**Figure 8i-k**). In summary, the nomograms integrating the HLMRGS and clinical characteristics showed exceptional predictive accuracy and clinical applicability in GBM patients.

### 3.7 Identification of potential therapeutic drugs for GBM patients with high HLMRGS and targeted prognostic genes

**Figure 9:**
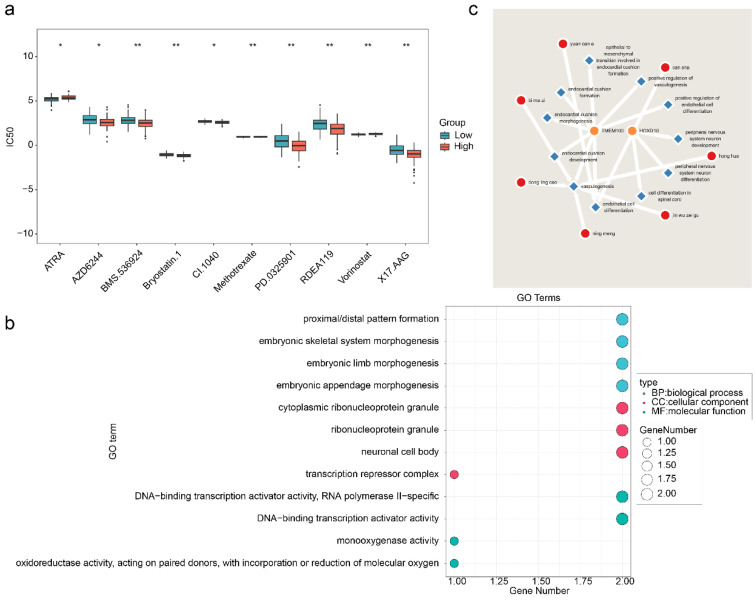
** Potential therapeutic drugs for GBM patients.** (a) Drug sensitivity analyses for the high- and low-risk groups. (b) GO enrichment analysis of prognostic genes. (c) Traditional Chinese medicines related to prognostic genetic and biological parameters associated with immune infiltration.

Analysis of the therapeutic drugs revealed significant differences in the IC50 values of the 12 drugs between the high- and low-risk groups, with the top 10 drugs showing the most significant changes (**Figure 9a**). Among these drugs, AZD6244 exhibited a lower IC50 in the high-risk group, suggesting increased sensitivity in patients with GBM with high HLMRGS, potentially enabling better therapeutic effects at lower doses. Conversely, ATRA had a lower IC50 in the low-risk group than in the high-risk group, indicating improved efficacy in patients with low HLMRGS. These variations may reflect differences in drug metabolism or mechanisms of action influenced by prognostic genes. These findings suggest that HLMRGs could modulate drug sensitivity, providing a basis for personalized drug therapy in GBM. This approach could optimize treatment plans on the basis of HLMRGS stratification and increase the therapeutic efficacy of targeted drugs.

In addition, enrichment analysis of the prognostic genes revealed 96 significant GO terms, including 86 BPs, 6 CCs, and 4 MFs. The top four significant entries, such as those related to proximal/distal pattern formation, were visualized (**Figure 9b**). Thirteen immune infiltration-related BPs were identified, including positive regulation of endothelial cell differentiation (**Table S5**). Chinese medicine predictions revealed that Can sha corresponded directly to HOXD10, whereas no direct matches were found for the other prognostic genes. However, a total of 7 Chinese medicines were predicted for all the immune infiltration-related BPs of prognostic genes, among which 2 Chinese medicines were predicted for endothelial cell differentiation, namely, can sha and Yuan can e, whereas 5 Chinese medicines were predicted for vasculogenesis, namely, Bi ma zi, Dong ling cao, Ning meng, Jin wu zei gu and Hong hua (**Figure 9c**). These medicines may exert therapeutic effects by targeting HLMRGs or their associated biological processes, suggesting their potential as therapeutic agents for patients with GBM.

## 4. Discussion

GBM is an aggressive and incurable brain tumor characterized by a low 5-year survival rate and a high recurrence rate 59. HLM is a histone modification associated with metabolic stress that plays a crucial role in immune regulation and the maintenance of homeostasis in diseases such as cancer. 24, 60. This study integrated scRNA-seq and bulk RNA-seq data to evaluate the prognostic significance of HLMRGs in GBM via bioinformatics approaches. A prognostic risk model was constructed on the basis of five key genes (SNCAIP, TMEM100, NLRP11, HOXC11, and HOXD10) to analyse the molecular regulatory mechanisms and immune characteristics associated with HLMRGs. The ultimate goal is to facilitate the development of targeted therapies for GBM and improve patient survival.

Our findings revealed elevated Kla levels in GBM, which were significantly associated with patient prognosis. These results align with previous studies reporting that increased Kla in gastric cancer and malignant melanoma correlates with poor prognosis, suggesting that Kla is a potential prognostic biomarker in these malignancies 24, 61. Furthermore, the high-risk group exhibited significantly higher infiltration scores for 16 distinct immune cell types, including activated dendritic cells, compared to the low-risk group. Notably, there was a discernible divergence in the immune microenvironment across the HLMRGS groups. These findings suggest that HLM may contribute to GBM progression by modulating the immune microenvironment. Recent studies support this notion, showing that lactate generation inhibitors can alleviate immunosuppression in the tumor microenvironment and potentially enhance CAR-T-cell efficacy in GBM treatment 20. Furthermore, PERK-driven glucose metabolism has been shown to promote the immunosuppressive activity of monocyte-derived macrophages (MDMs) through histone lactylation, and combining such approaches with immunotherapy may effectively inhibit GBM progression 62. In summary, these findings highlight the pivotal role of histone lactylation in the prognosis and immunotherapy of patients with GBM.

TCGA-GBM transcriptome data analysis revealed five prognostic genes (HOXC11, HOXD10, TMEM100, SNCAIP, and NLRP11) that are significantly overexpressed in GBM and are likely associated with its progression. The HOXC11 gene, a member of the homeobox gene family, plays a critical role in the morphogenesis of multicellular organisms 63. In GBM, a risk model incorporating HOXC11 has demonstrated the ability to predict patient prognosis 64. HOXC11 is overexpressed in colorectal cancer (CRC) and lung adenocarcinoma (LUAD) and is associated with poor patient prognosis. HOXC11 can regulate the chemotherapy resistance of CRC and increase the proliferation, migration and invasion of LUAD cells 63, 65. HOXD10, a member of the Abd-B homeobox gene family, has emerged as a detrimental prognostic biomarker in glioblastoma patients, aligning seamlessly with the conclusions drawn from the present investigation^66^. Previous research has demonstrated that miRNA-23a and miRNA-10b regulate GBM tumor invasion by targeting HOXD10 67, 68. TMEM100 is distributed across various cellular components and serves as a marker for the mitotic subtype of GBM, one of three primary subtypes, alongside invasive and intermediate types 69. Its downregulation has been implicated in nonleptomeningeal metastasis in patients with GBM 70. In addition to its role in GBM, TMEM100 serves as a prognostic marker in esophageal cancer, LUAD, and gastric cancer, where it is closely linked to immune cell infiltration 71-73. The overexpression of TMEM100 has been shown to inhibit the proliferation, invasion, and migration of esophageal squamous cell carcinoma cells 71.

Additionally, TMEM100 suppresses proliferation, migration, and invasion in prostate cancer, as well as migration and angiogenesis in colorectal cancer (CRC) cells 74, 75. SNCAIP is an intracellular prognostic marker in GBM that is involved in cytoplasmic inclusion body formation and neurodegeneration 76. SNCAIP duplication may promote the development of type 4 medulloblastoma by inducing PRDM6 77. Patients with metastatic clear cell renal cell carcinoma are more likely to present with SNCAIP alterations 78. NLRP11, a member of the NOD-like receptor protein family, is implicated in the proliferation and metastasis of LUAD 79, as well as in steroid-resistant multiple myeloma and retinoblastoma 80, 81. In summary, these five prognostic genes not only are prognostic markers of GBM but also may play important roles in the proliferation, migration, invasion, angiogenesis, immune infiltration and chemotherapy resistance of GBM.

GSEA identified the cytokine‒cytokine receptor interaction pathway as a key pathway distinguishing high- and low-risk groups in GBM. Previous investigations have elucidated that HOXD10 expression predominantly encompasses this pathway, with its role demonstrating variability—often even exhibiting opposing effects—across different stages of GBM progression and development.^66^. This pathway is critical for GBM growth and immune evasion 82. The differential roles of HOXD10 in various stages of GBM suggest that its high expression may modulate the activity of this pathway, exerting multifaceted effects on tumor progression. Th1-like CD4 T helper cells are critical for antitumour immunity and long-term survival in patients with GBM 83. Studies have shown that knocking down NLRP11 in Burkitt B lymphoma cells increases IFN-γ and IL-17A and that adenosine negatively regulates Th1 cell responses via NLRP11 84. These findings underscore the significant role of NLRP11 and immune cells in the tumor immune response.

In this study, immune cell infiltration analysis revealed significant differences in immune cell populations, particularly Th1 cells, between the high- and low-risk GBM groups, with these cells strongly correlated with prognostic genes. These results suggest that prognostic genes may regulate the GBM immune microenvironment by influencing immune cell activity and function, thereby affecting tumor progression and therapeutic responses. Understanding these relationships could facilitate the development of novel immunotherapy strategies to improve treatment outcomes in GBM patients. Interactions between microglia and Th1 cells have been reported to stimulate microglia-mediated antitumour effects in GBM 83. Grounded in cell trajectory analysis, prognostic genes are shown to modulate microglia-Th1 cell interactions through the alteration of their expression profiles, thereby assuming a critical role in the initiation and progression of GBM.

We know that GBM contains a variety of immune infiltrating cells^85^, immune-infiltrating cells play an important role in cancer proliferation and development^86^, ^87^. We know that glycolysis and lactate play an important role in tumor growth, while lactate enhances the expression of regulatory T cells and helps to defend against malignant cells, thereby escaping the attack of the immune system 88, 89. Studies have reported that high lactate can affect immune infiltrating cells, lactate regulates the metabolism of endogenous and adaptive immune cells through lactylation modification to form immunosuppression, and this possible mechanism needs to be explored 90, 91. Regulatory T cells (Tregs) play a crucial role in maintaining the immunosuppressive microenvironment, and lactate can promote tumorigenesis by regulating the lactatation of MOESIN and enhancing TGF-β signaling, thereby inducing the production of efficient Tregs 90. High concentrations of lactate can inhibit the secretion of proinflammatory cytokines by cytotoxic T lymphocytes (CTLS), lead to T cell dysfunction, and induce immunosuppression 90, 92, 93. Lactate-associated polarization of tumor-associated macrophages plays an important role in the immune escape of malignant tumors 90, 94. Lactate can recruit peripheral blood macrophages to infiltrate the tumor site, and then induce the expression of vascular endothelial growth factor and arginase 1 to influence polarization 94, 95. The level of lactate is closely related to immune signaling and contributes to the remodeling of the tumor environment 96, 97. High lactate can lead to tumor immune escape by impairing tumor surveillance by T or NK cells 36, 93.

Analysis of the scRNA-seq datasets revealed Micro as the predominant cell population in GBM, which is consistent with the findings of previous studies 98, 99. Microglia are essential for anti-GBM CD4+ T-cell responses and the inhibition of tumor growth 83. However, microglia and macrophages can also secrete cytokines and growth factors that contribute to immune evasion, tumor growth, and invasion 100.

Cytokines released by GBM cells can interact with microglia to increase glutamate levels in the tumor microenvironment, driving astrocyte scar formation, which in turn limits GBM growth 101. These findings highlight the dual role of microglia in GBM development and the tumor microenvironment. In light of the significant infiltration of microglia in GBM, which contributes to tumor progression, immunosuppression, and therapy resistance 99, it is a plausible speculation that prognostic genes may regulate microglial infiltration by manipulating their expression patterns. This modulation could have profound effects on GBM progression and treatment resistance.

This study identified five HLM-related prognostic genes with significant prognostic value in GBM, and the risk model constructed using these genes demonstrated strong and stable predictive performance for GBM prognosis. These findings provide a theoretical foundation and direction for early GBM diagnosis, treatment, and investigation of HLMRG mechanisms. However, this study has certain limitations. The molecular mechanisms underlying the expression of prognostic genes in GBM have not been explored. Additionally, the predictive power of the risk model and the clinical applicability of identified therapeutic drugs require further validation through large-scale, evidence-based research. Prospective studies are necessary to confirm these findings and to further elucidate the mechanisms by which HLM-related prognostic genes influence GBM progression and treatment outcomes.

## 5. Conclusion

In this study, histone lactylation was found to have an important effect on the clinical prognosis of GBM patients, and five HLM-related prognostic genes (SNCAIP, TMEM100, NLRP11, HOXC11, and HOXD10) were identified in GBM patients. The established prognostic model showed good prognostic ability for GBM patients. HLMRGs play important roles in cell cycle regulation, cell interactions, and immune cell infiltration and identify potential therapeutic drugs for GBM. These results provide a solid theoretical basis and valuable reference for the development of personalized treatment strategies and targeted drugs for GBM therapy.

## Supplementary Material

Supplementary figures and tables.
