# Supplementary material for: Identification and Validation of Prognostic Genes Related to Histone Lactylation Modification in Glioblastoma: An Integrated Analysis of Transcriptome and Single-cell RNA Sequencing
Source: J Cancer. 2025 Mar 21;16(7):2145–66. doi: 10.7150/jca.110646 (PMC12036088; doi:10.7150/jca.110646)
Supplement: Supplementary file 1 — Supplementary figures and tables. [file jcav16p2145s1.pdf]

Supplementary materials

Supplementary figures

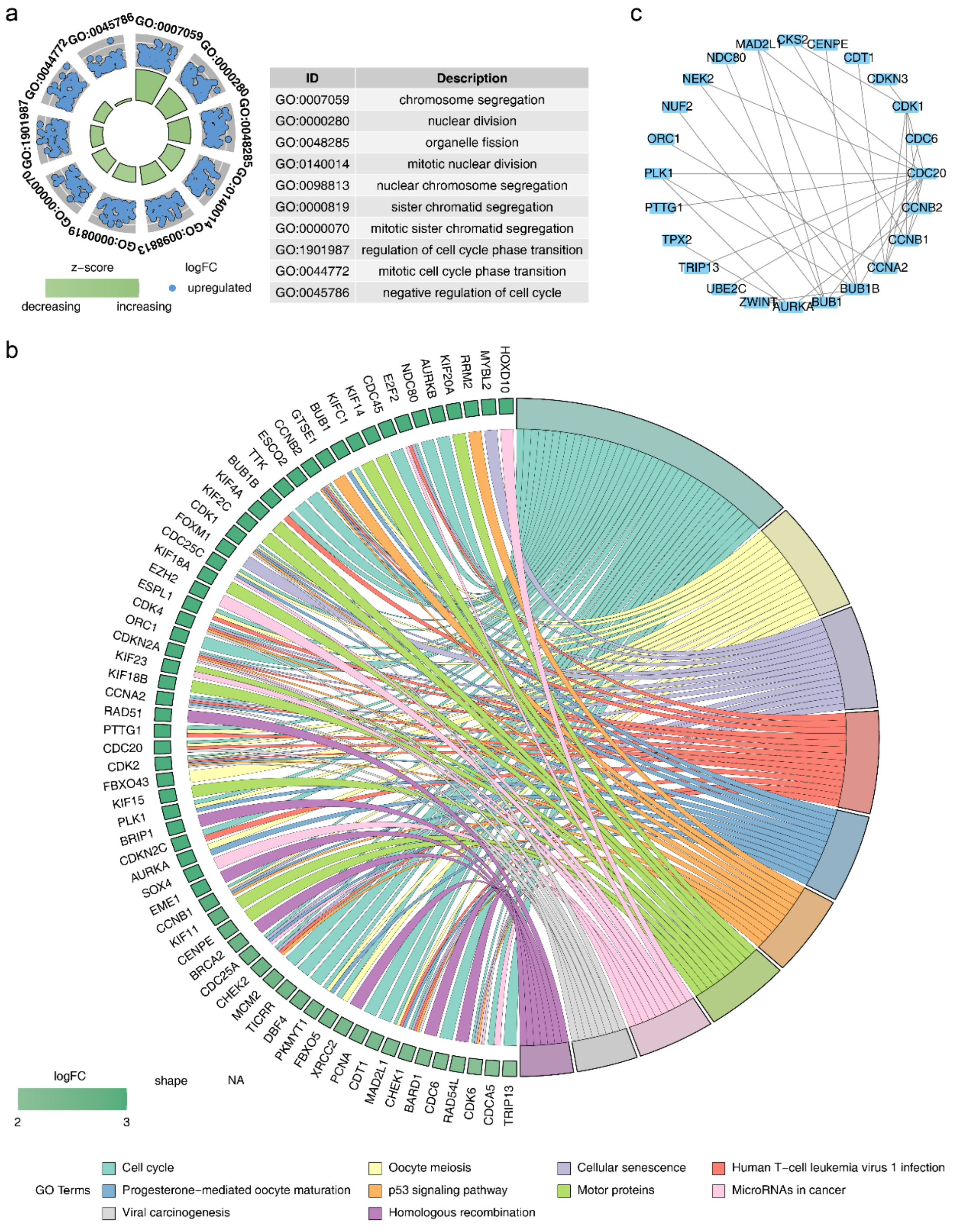

Figure S1 Enrichment analysis of candidate genes. (a) GO enrichment analysis of candidate genes. (b) KEGG enrichment analysis of candidate genes. (c) PPI network of candidate genes.

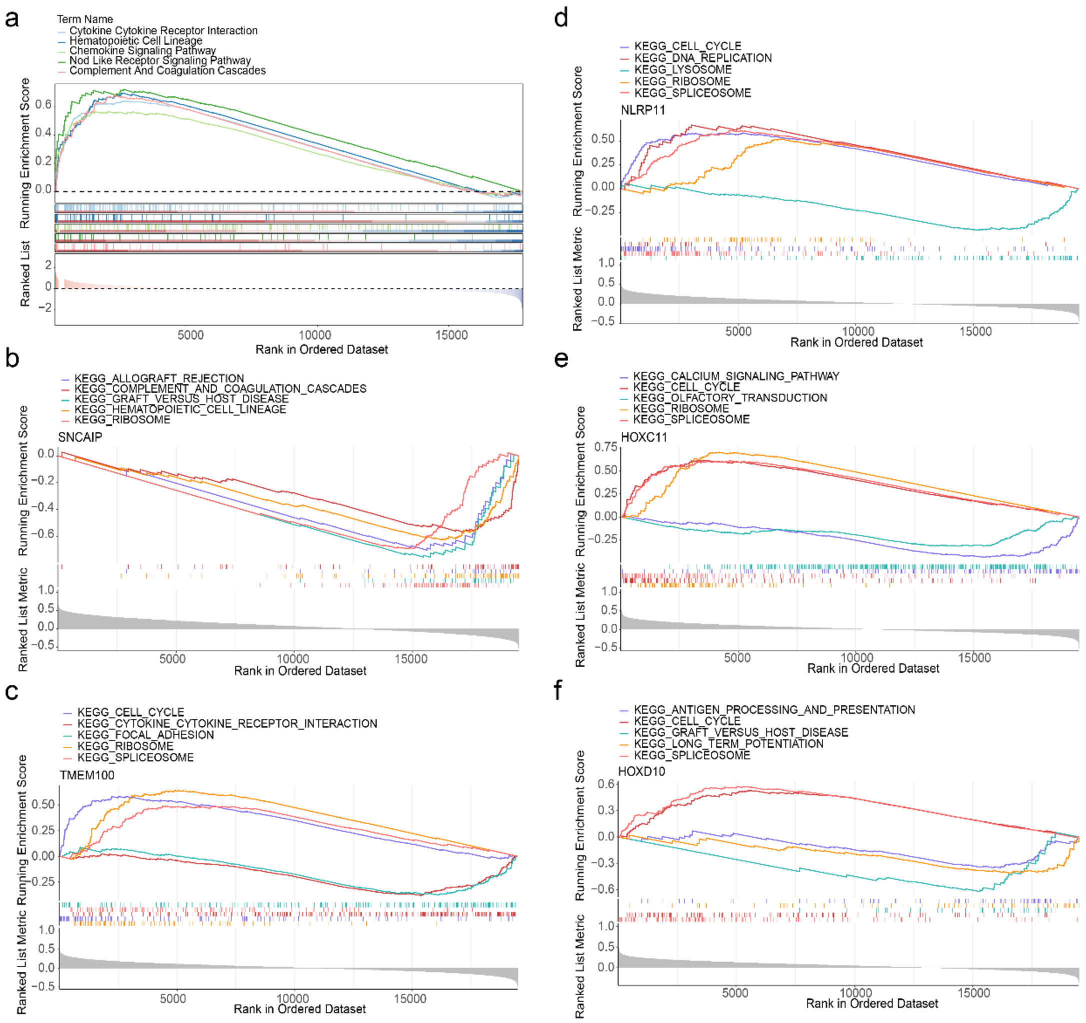

Figure S2 Results of enrichment analysis. (a) GSEA enrichment analysis of the TCGA-GBM dataset. (b) Enrichment analysis results for SNCAIP. (c) Enrichment analysis results for TMEM100. (d) Enrichment analysis results for NLRP11. (e) Enrichment analysis results for HOXC11. (f) Enrichment analysis results for HOXD10.

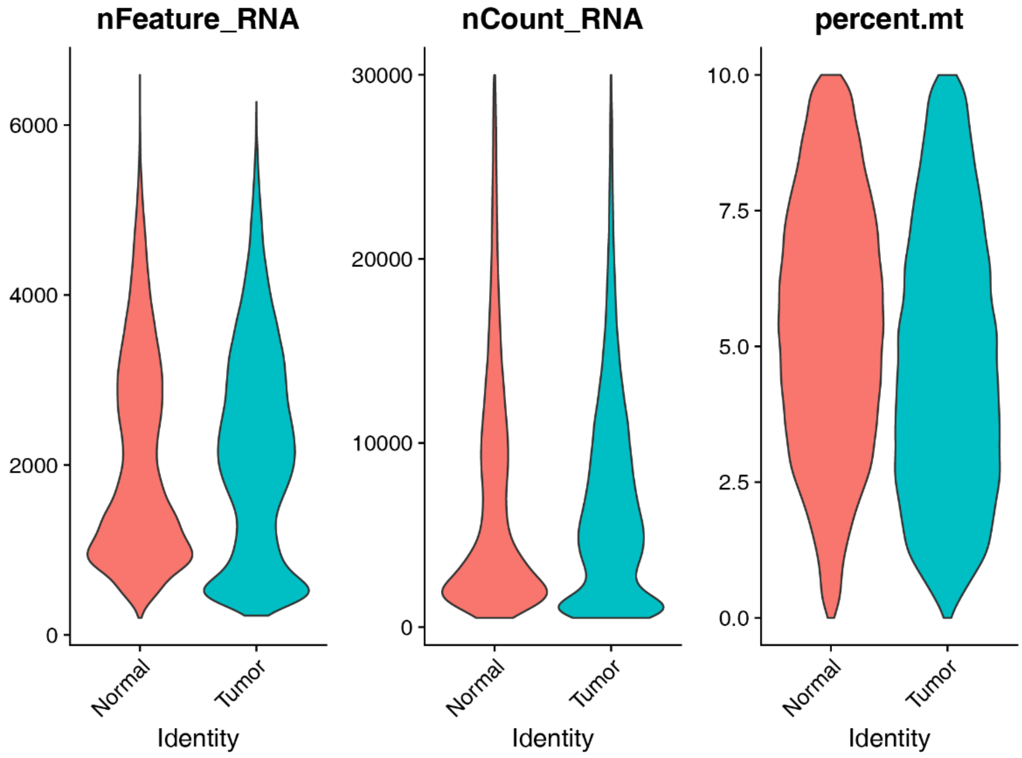

Figure S3 Screening of the scRNA-seq data.

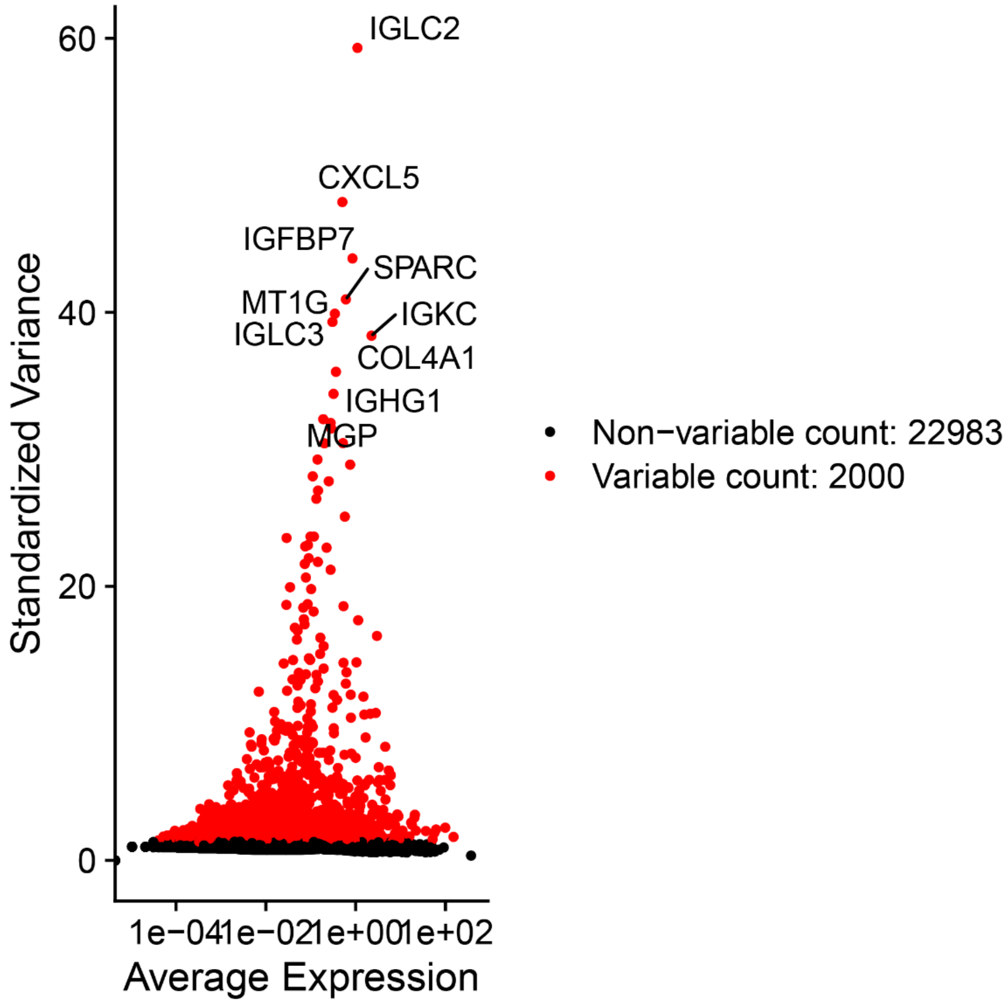

Figure S4 Screening for highly variable genes.

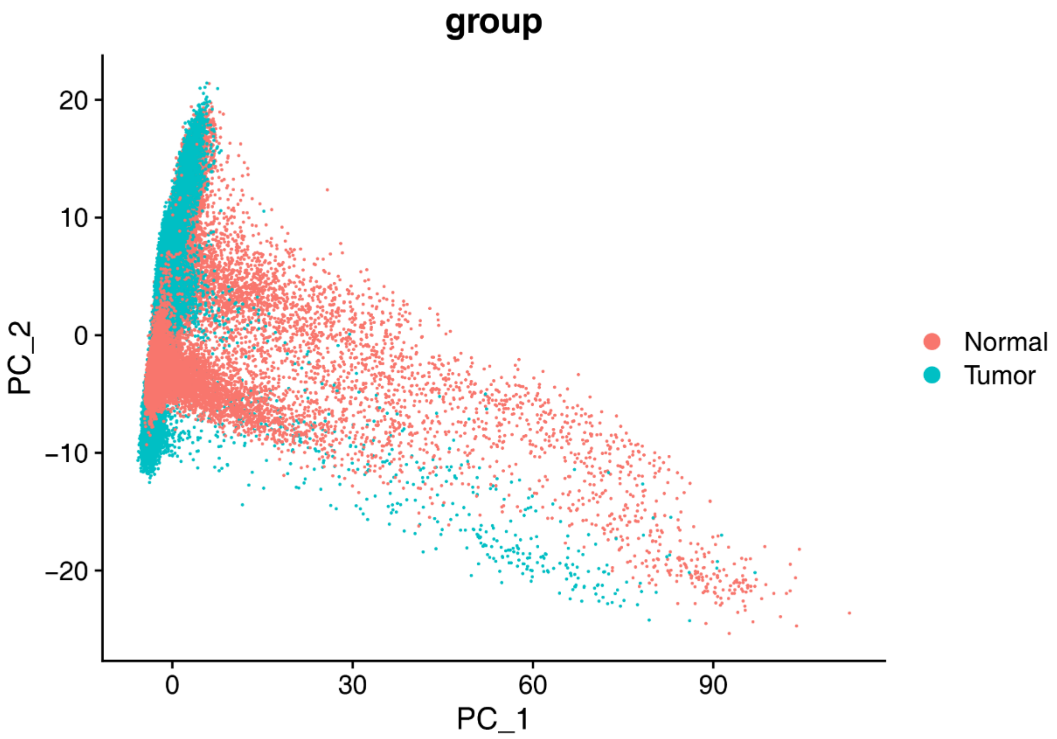

Figure S5 Results of PCA analyses.

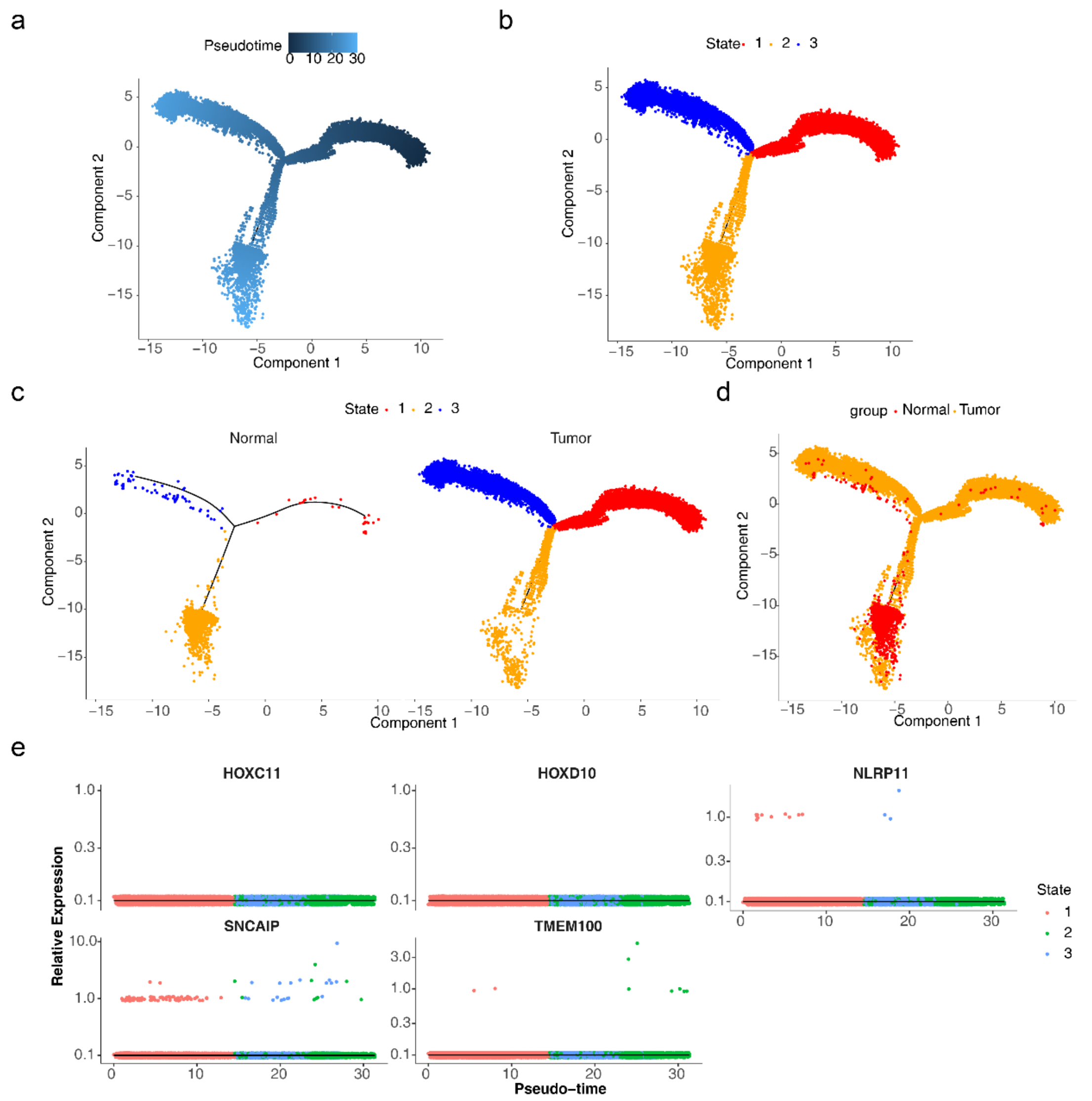

Figure S6 Results of pseudotiming analysis. (a) Cell track differentiation of Micro. (b) Differentiation states of Micro. (c) The major cell types in the GBM and normal groups after microdifferentiation. (d) The major cell types in the GBM and normal groups. (e) Prognostic gene expression in the micro at different states of differentiation.

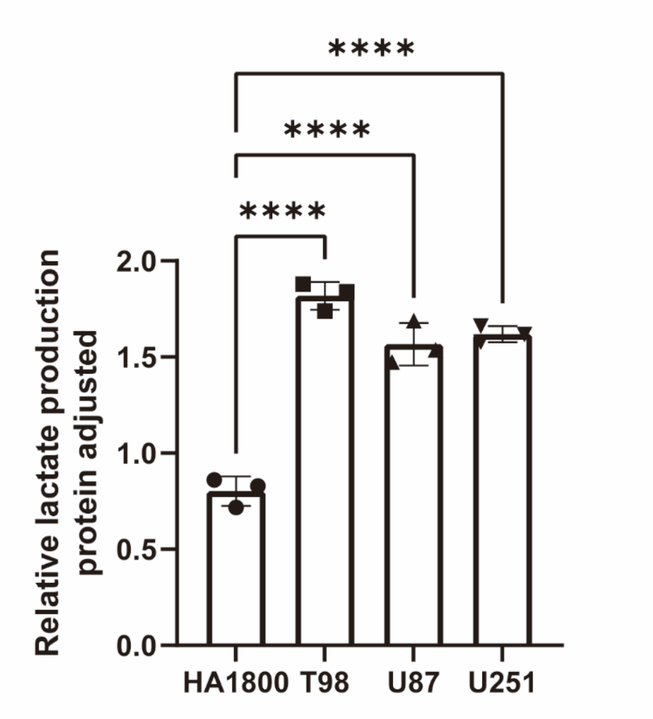

Figure S7 Results of lactate measurements.

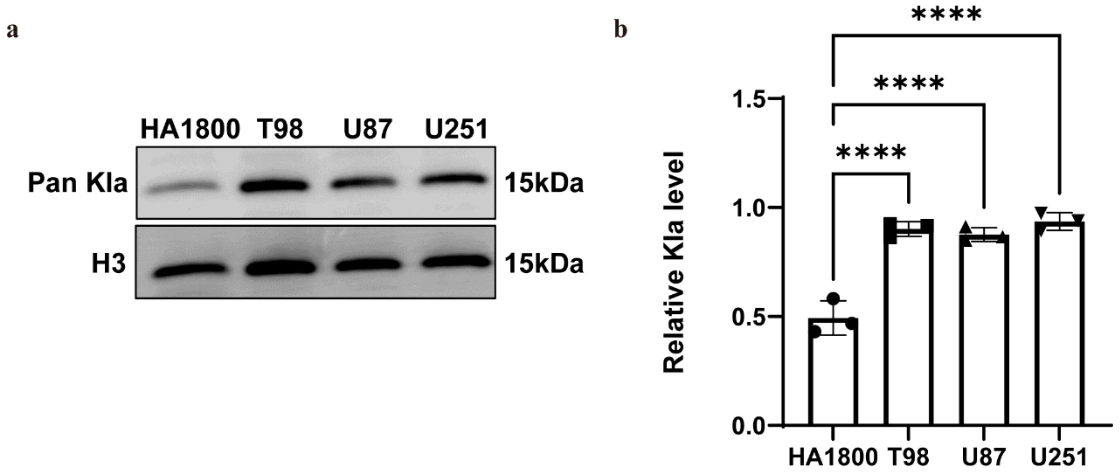

Figure S8 Results of Kla protein level measurements. (a) Western blot protein bands of Kla. (b) Quantitative Profiling of Kla Protein Expression Levels.

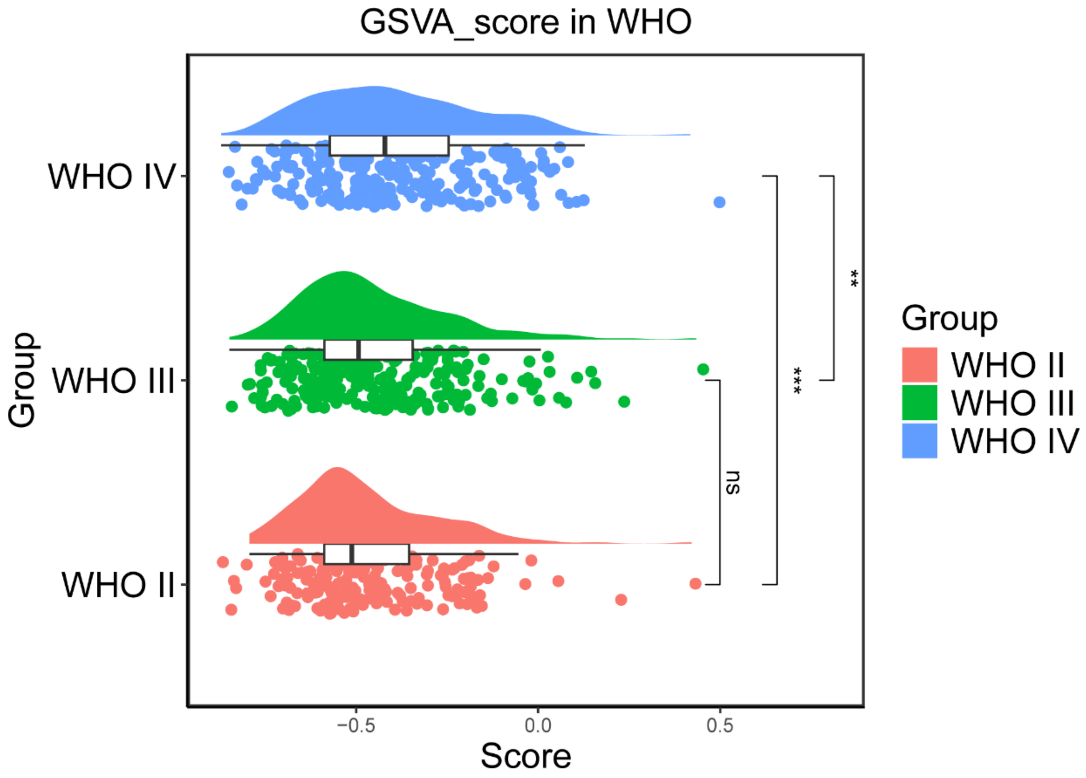

Figure S9 HLMRGs scores of different WHO grading samples

Supplementary tables

Table S1 Demographic and clinical characteristics of the study cohort.

ID	Gender	Age	WHO grading
Patient 1	F	69	2
Patient 2	M	23	2
Patient 3	M	39	2
Patient 4	M	45	2
Patient 5	M	29	2
Patient 6	F	59	2
Patient 7	F	53	2
Patient 8	F	50	2
Patient 9	M	46	2
Patient 10	M	37	2
Patient 11	F	17	1
Patient 12	F	58	2
Patient 13	M	38	2
Patient 14	M	55	2
Patient 15	F	44	4
Patient 16	F	53	4
Patient 17	M	55	4
Patient 18	M	65	4
Patient 19	F	59	4

Patient 20	M	68	4
Patient 21	M	46	4
Patient 22	M	62	4
Patient 23	M	65	4
Patient 24	M	62	4
Patient 25	M	69	4
Patient 26	M	50	4
Patient 27	F	64	4
Patient 28	M	45	4

Table S2 Results of the PH assumption test.

**Genes**	**P**
SNCAIP	0.337580111388179
HOXC11	0.630128848566583
HOXD10	0.558442254914382
TMEM100	0.367340258349072
NLRP11	0.509376809418571

Table S3 Results of the PH assumption test in the TCGA-GBM dataset.

**Factor**	**P**
risk score	0.321258781801055
age	0.119750637898457

Table S4 Results of the PH assumption test on the CGGA-GBM dataset.

**Factor**	**P**

MGMTp	0.321949139
PRS	0.163408358
Age	0.720859333

Table S5 Biological parameters related to immune infiltration.

ID	Description	BgRatio	pvalue	geneID
GO:0045603	positive regulation of endothelial cell differentiation	19/18870	0.005025	TMEM100
GO:2001214	positive regulation of vasculogenesis	12/18870	0.003176	TMEM100
GO:0003198	epithelial to mesenchymal transition involved in endocardial cushion formation	15/18870	0.003969	TMEM100
GO:0003272	endocardial cushion formation	28/18870	0.007398	TMEM100
GO:0003203	endocardial cushion morphogenesis	41/18870	0.010818	TMEM100
GO:0003197	endocardial cushion development	52/18870	0.013704	TMEM100
GO:0001570	vasculogenesis	83/18870	0.021802	TMEM100
GO:0045446	endothelial cell differentiation	124/18870	0.032431	TMEM100
GO:0045603	positive regulation of endothelial cell differentiation	19/18870	0.005025	TMEM100
GO:0021515	cell differentiation in spinal cord	50/18870	0.01318	HOXD10
GO:0048934	peripheral nervous system neuron differentiation	15/18870	0.003969	HOXD10
GO:0048935	peripheral nervous system neuron development	15/18870	0.003969	HOXD10
GO:0045603	positive regulation of endothelial cell differentiation	19/18870	0.005025	TMEM100
